# Differentially Expressed Genes in *Bordetella pertussis* Strains Belonging to a Lineage Which Recently Spread Globally

**DOI:** 10.1371/journal.pone.0084523

**Published:** 2014-01-08

**Authors:** Daan de Gouw, Peter W. M. Hermans, Hester J. Bootsma, Aldert Zomer, Kees Heuvelman, Dimitri A. Diavatopoulos, Frits R. Mooi

**Affiliations:** 1 Department of Pediatrics, Laboratory of Pediatric Infectious Diseases, Radboud University Medical Centre, Nijmegen, The Netherlands; 2 Nijmegen Institute for Infection, Inflammation and Immunity, Radboud University Medical Centre, Nijmegen, The Netherlands; 3 Netherlands Centre for Infectious Disease Control, National Institute for Public Health and the Environment (RIVM), Bilthoven, The Netherlands; Universidad Nacional de La Plata., Argentina

## Abstract

Pertussis is a highly contagious, acute respiratory disease in humans caused by the Gram-negative pathogen *Bordetella pertussis*. Pertussis has resurged in the face of intensive vaccination and this has coincided with the emergence of strains carrying a particular allele for the pertussis toxin promoter, *ptxP3*, which is associated with higher levels of pertussis toxin (Ptx) production. Within 10 to 20 years, *ptxP3* strains have nearly completely replaced the previously dominant *ptxP1* strains resulting in a worldwide selective sweep. In order to identify *B. pertussis* genes associated with the selective sweep, we compared the expression of genes in *ptxP1* and *ptxP3* strains that are under control of the *Bordetella* master virulence regulatory locus (*bvgASR*). The BvgAS proteins comprise a two component sensory transduction system which is regulated by temperature, nicotinic acid and sulfate. By increasing the sulfate concentration, it is possible to change the phase of *B. pertussis* from virulent to avirulent. Until recently, the only distinctive phenotype of *ptxP3* strains was a higher Ptx production. Here we identify additional phenotypic differences between *ptxP1* and *ptxP3* strains which may have contributed to its global spread by comparing global transcriptional responses under sulfate-modulating conditions. We show that *ptxP3* strains are less sensitive to sulfate-mediated gene suppression, resulting in an increased production of the vaccine antigens pertactin (Prn) and Ptx and a number of other virulence genes, including a type III secretion toxin, Vag8, a protein involved in complement resistance, and *lpxE* involved in lipid A modification. Furthermore, enhanced expression of the vaccine antigens Ptx and Prn by *ptxP3* strains was confirmed at the protein level. Identification of genes differentially expressed between *ptxP1* and *ptxP3* strains may elucidate how *B. pertussis* has adapted to vaccination and allow the improvement of pertussis vaccines by identifying novel vaccine candidates.

## Introduction

*Bordetella pertussis* is the major causative agent of whooping cough or pertussis, a highly contagious, acute respiratory disease in humans. Before the introduction of vaccination in the 1950s, pertussis was a leading cause of infant death throughout the world [1] Although extensive immunization campaigns have significantly reduced pertussis-caused infant mortality worldwide, infections with *B. pertussis* still pose an important health burden, with an annual infection rate of 1–9% in some highly vaccinated populations [2] and an estimated 195,000 deaths worldwide [3]. Significantly, pertussis notifications have increased during the last two decades, especially in adolescents and adults. This increase of pertussis infections in older age categories represents significant health risks, as they are a source of *B. pertussis* transmission to unvaccinated infants for whom pertussis is a severe, life threatening, disease [4]–[6]. In 2010 and 2012 particularly large outbreaks of pertussis were observed in several countries [7]–[9]. Suggested causes for the increase in pertussis include increased awareness, improved diagnostics, suboptimal vaccines, waning immunity, and pathogen adaptation [10], [11].

Studies performed in several countries provide evidence that *B. pertussis* has diverged antigenically from vaccine strains and has increased expression of pertussis toxin (Ptx) and the type III secretion system (T3SS) effector toxin BteA [12], [13]. In the Netherlands, the pertussis resurgence has coincided with the emergence of strains that carry a particular allele of the *ptx* operon promoter, *ptxP3*, which is associated with higher levels of Ptx expression [13]. Within a timeframe of 10 years, *ptxP3* strains have nearly completely replaced the previously dominant *ptxP1* strains. Significantly, *ptxP3* strains have spread globally, as they have emerged in several countries in Europe [13]–[17], North America [18], South America [19], and Australia [20]. Phylogenetic analyses showed that *ptxP3* strains evolved from *ptxP1* strains, possibly in the 1970s [21], [22]. A recent comparative genomic analysis of two newly emerged *ptxP3* strains, two contemporary *ptxP1* strains, and two pre-vaccination strains identified single nucleotide polymorphisms (SNPs) in a number of pathogenicity-associated genes, as well as differences in gene inactivation and reactivation [23]. Thus far, very little information is available on phenotypic differences between *ptxP1* and *ptxP3* strains, such as the regulation and expression of virulence genes.

*B. pertussis* produces multiple toxins, including Ptx, adenylate cyclase toxin (ACT), T3SS effectors, dermonecrotic toxin (DNT), and tracheal cytotoxin (TCT) that, together with other virulence factors, facilitate within-host survival by manipulating many aspects of the human immune system, including the complement system, phagocytosis, immune cell recruitment, and antibody responses (reviewed in [24], [25]. The expression of most of these virulence factors is regulated by the activity of BvgS and BvgA, which form a typical two-component regulatory system, and the repressor protein BvgR which is expressed from the same *BvgASR* locus [26] (reviewed in [27]). Several external factors have been identified that can modulate the activity of this system, which results in a spectrum of different Bvg-phases that affect bacterial virulence. For instance, low temperature, and increasing concentrations of nicotinic acid or sulfate have all been shown to facilitate the transition from virulent (Bvg^+^) through intermediate (Bvg^i^) to nonvirulent (Bvg^−^) bacteria [28], [29].

Until recently, the only distinctive phenotypes described for *ptxP3* strains was a higher Ptx production and enhanced respiratory colonization [13], [30]. Here we identify additional phenotypic differences between *ptxP1* and *ptxP3* strains by comparing global transcriptional responses under sulfate-modulating conditions. We show that *ptxP3* strains are less sensitive to sulfate-mediated suppression, which is associated with an increased production of a number of virulence genes, including Ptx, a type III secretion toxin, Vag8, a protein involved in complement resistance [31], and *lpxE* involved in lipid A modification. Identification of genes differentially expressed between *ptxP1* and *ptxP3* strains may elucidate how *B. pertussis* has adapted to vaccination and allow the improvement of pertussis vaccines.

## Results

To gain more insight into the mechanisms underlying the emergence of *B. pertussis ptxP3* strains, we investigated differences in global gene expression between a representative *ptxP1* and *ptxP3* strain. Strains B1920 (*ptxP1*) and B1917 (*ptxP3*) were cultured *in vitro* in the presence of low (<0.02 mM), medium (5 mM) or high (50 mM) MgSO_4_. Our rationale for selecting MgSO_4_ is that free sulfate plays an important role in regulating the expression of a number of *B. pertussis* virulence genes through the *bvg* locus [28]. However, as sulfate may also affect expression of *B. pertussis* genes independent of the BvgASR system, we used the following definition of sulfate-regulated gene categories: high sulfate repressed (HSR) genes, high sulfate induced (HSI) genes, medium sulfate repressed (MSR) genes, and medium sulfate induced (MSI) genes. HSR and HSI genes were defined as those genes which were at least three-fold down- or upregulated in the presence of 50 mM sulfate compared to low sulfate, respectively. Similarly, MSI or MSR genes were defined as genes which were significantly up- or downregulated respectively by at least three-fold when bacteria were grown in the presence of 5 mM sulfate compared to low sulfate. The majority of the classical Bvg^+^, Bvg^−^, and Bvg^i^ genes are a subset of the HSR, HSI, and MSI genes respectively. MSR genes have, to the best of our knowledge, not been defined before.

### Validation of sulfate-induced *BvgASR*-modulation

**Figure 1:**
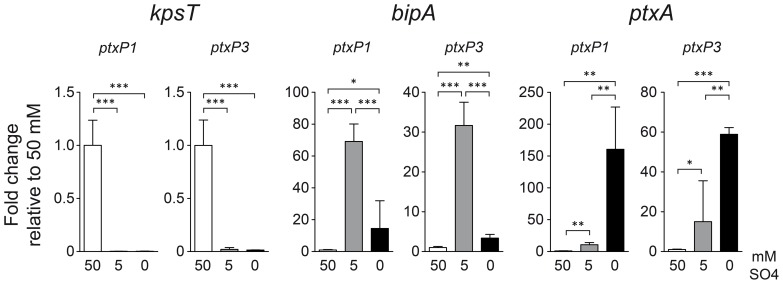
Sulfate-mediated modulation in *B. pertussis* strain B1920 (*ptxP1*) and B1917 (*ptxP3*). Sulfate was added to the culture medium to induce a high (50 mM), medium (5 mM), and low (<0.02 mM, represented as 0 mM on the x-axis) sulfate conditions. qRT-PCR data shows the relative expression level of *kpsT*, *bipA*, and *ptxA* expressed as fold changes relative to the high sulfate condition, with the values being the mean of four biological replicate cultures. Asterisks indicate a statistically significant difference between the groups as determined by Student's t-test with Welch's correction: * *P* value <0.05, ** *P* value <0.005, *** *P* value <0.0005.

To confirm the modulating effect of sulfate on Bvg-associated genes for the two selected strains, qPCR was performed on *kpsT*, *bipA*, and *ptxA*, coding for KpsT, a protein involved in capsule biosynthesis, the outer membrane ligand binding protein BipA, and the PtxA (or S1) subunit. The *kpsT, bipA*, and *ptxA* genes were chosen as they have previously been shown to be optimally expressed *in vitro* under Bvg^−^, Bvg^i^, and Bvg^+^ conditions, respectively [32], [33]. Here, we used them to represent HSI, MSI, and HSR genes, respectively. qPCR analysis showed a strong correlation between sulfate concentration and expression of *ptxA, bipA* and *kpsT*, thus demonstrating the classical sulfate-dependent expression phenotype in both strains (Figure 1).

### Sulfate-dependent transcriptional responses in a *ptxP1* and *ptxP3* strain

In order to identify HSR, HSI, MSI, and MSR genes, we performed global transcriptional analysis on *B. pertussis* strains B1920 (*ptxP1*) and B1917 (*ptxP3*) in the presence of low, medium, and high sulfate. The expression levels of all genes for both strains can be found in Table S7. To identify gene categories enriched for HSR, HSI, MSI, and MSR genes, genes were aggregated based on their function and predicted subcellular localization.

#### High sulfate repressed genes

**Figure 2:**
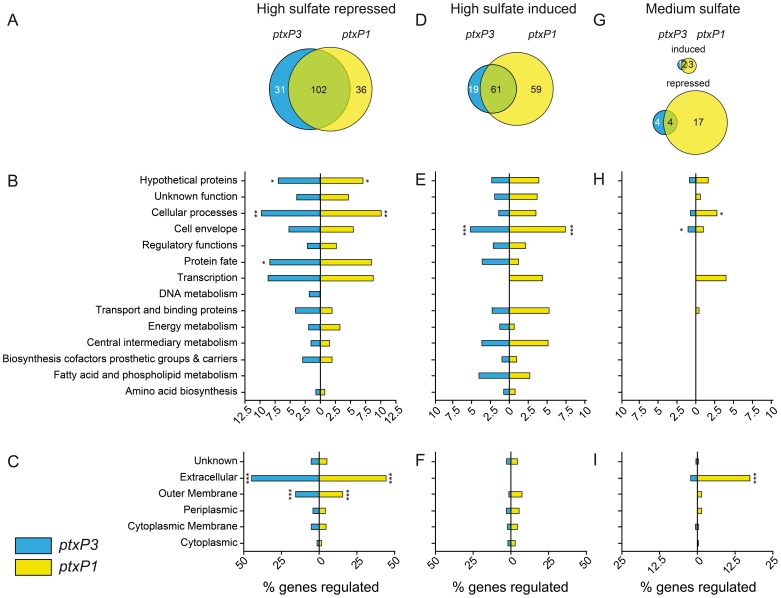
Functional categorization of sulfate-regulated genes in *B. pertussis* strain B1920 (*ptxP1*) and B1917 (*ptxP3*). Venn diagrams show overlapping and unique sulfate-regulated genes that are at least 3-fold downregulated under high sulfate conditions relative to the low sulfate condition (A; high sulfate repressed (HSR)), 3-fold upregulated under high sulfate conditions relative to the low sulfate condition (D; high sulfate induced (HSI)), 3-fold up- or down-regulated under medium sulfate conditions relative to the low sulfate condition (medium sulfate induced (MSI) and repressed (MSR)). HSR, HSI, and MSR genes were grouped by functional categories (B, E, and H) and PSORTb-predicated subcellular localization (C, F, and I). Data are expressed as the percentage that is sulfate-regulated among all annotated genes in each class. Asterisks indicate statistically significant enrichment of sulfate-regulated genes in a certain class as determined by Fisher's exact test. * *P* value <0.05, ** *P* value <0.005, *** *P* value <0.0005.

Comparative analysis identified 138 and 133 HSR genes in the *ptxP1* and *ptxP3* strain, respectively (Figure 2A–C and Table S3, see Table S4 for strain-specific HSR and HSI genes). Of these genes, a large number (102 genes, corresponding to 74% of all HSR genes) was shared between the two strains, while 31 (23%) and 36 (28%) were repressed by high sulfate only in the *ptxP3* or the *ptxP1* strain, respectively (Figure 2). Functional class categorization of these 31 *ptxP3*-specific HSR genes showed enrichment for genes encoding ABC-transporters known to be involved in the uptake of small molecules, including sulfate.

Interestingly, based on the annotated Tohama I sequence and BLAST searches, 9 of the 30 genes predicted to be involved in sulfate-uptake and metabolism were *ptxP3*-specific HSR genes (N = 9, 29%, P<0.001, Table 1, Table S4). These results strongly suggested that sulfate uptake and metabolism are different between *ptxP1* and *ptxP3* strains. Besides sulfate genes, 22 other genes were identified as *ptxP3* strain-specific HSR genes (Table S4). Perhaps most interesting were the virulence-associated *fim3* and *lpxE* genes, encoding the major fimbrial subunit of serotype 3 fimbriae and a lipid A-1 phosphatase with LPS modifying properties, respectively (Table S4). Although *fim3* was also suppressed at the high sulfate concentration in the *ptxP1* strain, the value did not reach statistical significance. qPCR analysis supported the observation that the *fim3* gene is a HSR gene exclusively in the *ptxP3* strain (Figure S1).

**Table 1:** Transcriptional regulation of sulfate genes in *B. pertussis* strain B1920 (*ptxP1*) and B1917 (*ptxP3*).

General gene information				HSR Fold change		Fold difference between strains (*ptxP3/ptxP1*)		
ORF	Gene	Product	Predicted localization	*ptxP3*	*ptxP1*	0 mM	5 mM	50 mM
BB0537		sulfurtransferase	Un	†	†	†	†	†
BP0380		Sodium:sulfate symportert	Cm	−1.0	1.5	1.0	1.3	1.6
BP0958	*cysM*	cysteine synthase B	C	−1.0	−1.1	−1.0	1.0	−1.2
BP0966	*sbp*	sulfate-binding protein precursor	P	7.3^*^	†	9.3	3.1	†
BP0967	*cysT*	sulfate transport system permease protein (Pseudogene)	Cm	4.7^*^	†	4.2	†	†
BP0968	*cysW*	sulfate transport system permease protein (Pseudogene)	Un	†	†	†	†	†
BP0969	*cysA*	sulfate transport ATP-binding protein	Cm	†	†	†	†	†
BP0970	*cysH*	phosphoadenosine phosphosulfate reductase (Pseudogene)	P	†	†	†	†	†
BP0970A	*cysD*	sulfate adenylyltransferase subunit 2	C	†	†	†	†	†
BP0971	*cysN*	sulfate adenylyltransferase subunit 1 (Pseudogene)	Un	†	†	†	†	†
BP1055	*cysG*	siroheme synthase	C	4.7^*^	†	3.2	†	†
BP1362		amino-acid ABC transporter, ATP-binding protein	Cm	3.6^*^	†	3.4	†	†
BP1363		amino-acid ABC transporter, permease protein	Cm	6.2^*^	†	4.4	3.2	†
BP1364		amino-acid ABC transporter, periplasmic amino acid-binding protein	P	4.2^*^	1.8	2.7	1.7	1.1
BP1908	*cysS*	cysteinyl-tRNA synthetase	C	−1.5	−1.3	−1.5	−1.9	−1.3
BP2416	*cysB*	LysR-family transcriptional regulator	C	2.2^*^	−1.3	2.7^*^	1.2	−1.1
BP2816		ABC transport protein, ATP-binding component	Cm	3.5^*^	1.2	3.1^*^	1.1	1.0
BP3432	*cysI*	sulfite reductase	C	4.5^*^	2.2	2.7	1.4	1.3
BP3434		exported protein	Cm	3.7^*^	†	3.8^*^	1.6	1.4
BP3455		taurine dioxygenase	C	†	†	†	†	†
BP3674		periplasmic solute-binding protein	P	†	†	†	†	†
BP3675		ABC transport system permease protein	Cm	†	†	†	†	†
BP3676		ABC transporter ATP-binding protein	Cm	†	†	†	†	†
BP3701		exported protein	Un	†	−23.9^*^	†	†	−4.1^*^
BP3702	*lpxL1*	GntR-family transcriptional regulator	C	†	−14.2^*^	†	†	†
BP3703	*lpxL2*	transport system permease protein	Cm	†	−18.9^*^	†	†	−4.0^*^
BP3705		ABC transporter ATP-binding protein	Cm	†	−16.8^*^	†	†	−2.9
BP3854		ABC transporter ATP-binding protein	Cm	†	†	†	†	†
BP3855		periplasmic protein	P	†	†	†	†	†
BP3856		taurine catabolism dioxygenase	C	†	†	†	†	†

Abbreviations; C, cytoplasmic; Cm, cytoplasmic membrane; FC, fold change; HSR, high sulfate repressed; ORF, open reading frame; P, periplasmic; Un, unknown. The columns with fold change difference show the ratio of absolute expression (*ptxP3*/*ptxP1*) under low (0 mM), intermediate (5 mM), and high (50 mM) sulfate conditions. Asterisks indicate a statistically significant difference. † indicates that the gene was excluded because signal intensity was ≤500 in both the conditions compared. Genes above the dotted line are genes that annotated as genes involved in sulfate metabolism.

In addition to the *ptxP3-*specific genes, we also identified 36 HSR genes which were repressed by high sulfate only in the *ptxP1* strain (Table S4). Although these 36 genes were not significantly enriched for any functional class, three virulence-associated genes were identified, namely the T3SS gene (*bscO*), an autotransporter (*bapC*), and a TonB-dependent receptor for iron transport (*bfrE*) (Table S4). However, it should be noted that all three genes showed a HSR phenotype in the *ptxP3* strain but were excluded because the microarray signal intensity was below 500.

#### High sulfate induced genes

Larger differences between the two strains were found when HSI genes were compared. Eighty and 120 high-sulfate induced genes were identified in the *ptxP3* and *ptxP1* strain, respectively (Figure 2D-F and Table S3), of which 61 (51%) were shared. Of the non-shared genes, 19 (24%) and 59 (49%) were unique for the *ptxP3* and *ptxP1* strain, respectively. Whilst the *ptxP3* strain-specific HSI genes were not significantly enriched for any functional category or subcellular localization, the 59 *ptxP1-*specific genes were significantly enriched for ABC-transporters involved in the transport of cyanate/nitrate, branched-chain amino acids, and capsular polysaccharides (N = 13, 22%, P<0.001, see Table S4). These data show that the HSI gene repertoire is highly different between the *ptxP1* and *ptxP3* strain.

#### Medium-sulfate regulated genes

Culturing *B. pertussis* in the presence of medium sulfate levels allowed us to identify both MSI and MSR genes by comparing the transcriptional levels to low sulfate conditions. This analysis yielded five and three MSI genes in the *ptxP1* and *ptxP3* strain, respectively, of which two were medium-sulfate induced in both strains (Figure 2G, Table 2, and Table S5). Despite the relatively low number of identified MSI genes as compared to the number of HSR and HSI genes, the MSI genes were significantly enriched for genes encoding cell envelope proteins in both the *ptxP3* (P = 0.027) and the *ptxP1* (P = 0.017) strain.

**Table 2:** Medium-sulfate regulated genes in *B. pertussis* strain B1920 (*ptxP1*) and B1917 (*ptxP3*).

General gene information				MSR Fold change		Fold difference between strains (*ptxP3/ptxP1*)		
ORF	Gene	Product	Predicted localization	*ptxP3*	*ptxP1*	0 mM	5 mM	50 mM
Genes upregulated by 5 mM sulfate								
BP1112	*bipA*	outer membrane ligand binding protein	Om	−3.3^*^	−4.4^**^	−3.1	1.0	†
BP2923		lipoprotein	Un	−3.9^*^	−4.9^*^	†	−1.3	−1.6
BP0713		membrane protein (Pseudogene)	Un	−3.8^*^	−4.1^*^	†	1.6	1.8
BP0468		AsnC-family transcriptional regulator	C	−1.4	−3.7^*^	2.5	−1.0	−1.1
BP0903		membrane protein	Cm	−1.9	−3.6^*^	†	−4.1^*^	†
BP1311		membrane protein	Cm	−2.4^*^	−3.5^*^	1.4	−1.0	1.2
Virulence genes downregulated by 5 mM sulfate								
BP3783	*ptxA*	pertussis toxin subunit 1 precursor	E	3.7^*^	6.3^*^	−1.3	1.5	1.1
BP3784	*ptxB*	pertussis toxin subunit 2 precursor	E	1.6	6.2^*^	−1.0	4.0^*^	†
BP3785	*ptxD*	pertussis toxin subunit 4 precursor	E	1.1	4.4^*^	1.0	4.0^*^	†
BP3787	*ptxC*	pertussis toxin subunit 3 precursor	E	1.0	3.2^*^	−1.0	3.2^*^	†
BP0499	*btcA*	t3ss chaperone	P	3.0^*^	4.5^*^	1.1	1.5	1.4
BP0500	*bteA*	t3ss toxin	E	1.8	3.3^*^	1.2	2.3	†
BP2234	*brpL*	RNA polymerase sigma factor	C	2.8^*^	7.1^*^	−1.5	1.8	†
BP2244	*bscO*	type III secretion protein	C	1.8	3.2^*^	−1.3	†	†
BP2261	*bcrD*	type III secretion pore protein	Cm	1.6	3.0^*^	−1.9	1.1	†
BP2265		type III secretion chaperone	Cm	4.6^*^	3.2^*^	1.1	−1.4	†
BP2315	*vag8*	autotransporter	E	1.4	3.6^*^	1.4	3.9^*^	†
BP2738	*bapC*	autotransporter (pseudogene)	E	1.4	3.7^*^	−1.2	†	†
BP2674	*fimX*	fimbrial protein	E	2.0^*^	3.0^*^	1.7	2.9	†
Other genes downregulated by 5 mM sulfate								
BP0398	*lmgB*	glycosyl transferase	Cm	3.2^*^	1.6	1.2	†	†
BP0399	*lmgA*	glycosyl transferase	Cm	3.1^*^	1.5	−1.0	−2.0	†
BP1252		exported protein	Un	3.6^*^	2.4^*^	1.1	−1.3	†
BP1286		conserved hypothetical protein	Un	3.1^*^	1.1	7.2^*^	2.5	†
BP1487	*smoM*	periplasmic solute-binding protein	P	2.1^*^	3.4^*^	−1.2	1.4	−1.1
BP2141		exported protein	Un	3.4^*^	5.1^*^	−2.0	−1.3	†
BP2147		exported protein	Un	1.9	3.0^*^	1.3	2.1	†
BP2486		exported protein	Cm	4.4^*^	4.0^*^	−1.6	†	†
BP2925		conserved hypothetical protein	C	1.2	4.0^*^	−1.6	1.9	†
BP2926		conserved hypothetical protein	Un	1.5	3.4^*^	−1.5	1.7	†
BP3095	*modB*	molybdate-binding periplasmic protein precursor	P	−1.1	3.4^*^	1.4	5.1^*^	1.9
BP3405	*ompQ*	outer membrane porin protein OmpQ	Om	1.3	3.6^*^	−1.1	2.4	†

Abbreviations; C, cytoplasmic; Cm, cytoplasmic membrane; E, extracellular; FC, fold change; MSR, medium sulfate repressed; Om, outer membrane; ORF, open reading frame; P, periplasmic; Un, unknown. The columns with fold change difference show the ratio of absolute expression (*ptxP3*/*ptxP1*) under low (0 mM), intermediate (5 mM), and high (50 mM) sulfate conditions. * Asterisks indicate a statistically significant difference. ** indicate a fold-change and statistically significant difference as determined by qPCR (see Figure 1). † indicates that the gene was excluded because the microarray signal intensity was ≤500 in both the conditions compared.

Larger differences were found when MSR genes were compared, as we identified twenty-one and eight MSR genes in the *ptxP1* and *ptxP3* strain, respectively. Of these genes, only four were shared between the two strains (Figure 2G, Table 2, and Table S5). In the MSR category, we found pronounced differences between the *ptxP1* and *ptxP3* strains in genes involved in cellular processes (a category which included most virulence factors) and genes encoding extracellular proteins (Figure 2H & I, Table 2). In particular, several known virulence genes were repressed only in the *ptxP1* strain under medium sulfate conditions, including two autotransporters (*vag8* and *bapC*), five T3SS genes (*btcA*, *bteA*, *bscO*, *bcrD*, and BP2265), and three *ptx* genes (*ptxB*, *ptxC*, *ptxD*) (Table 2 and Table S5). This strain-specific suppression of virulence genes under medium sulfate conditions became even more pronounced at a fold change cut-off of ≥2, at which five autotransporters, twelve T3SS, and seven *ptx* genes were suppressed in the *ptxP1* strain compared to the *ptxP3* strain (Table S5). Taken together, these data suggest that the *ptxP3* strain is less sensitive to sulfate-mediated modulation of virulence gene expression.

### Absolute differences in sulfate-dependent gene expression

Above, we assessed the effect of sulfate concentration on transcriptional up or down regulation within each strain. Whilst this approach is useful to identify strain-specific sulfate-regulated genes, it does not provide insight into differences between these strains in absolute terms. Therefore, we also compared absolute transcription levels *between* the *ptxP1* and *ptxP3* strain under low, medium, and high sulfate conditions.

**Figure 3:**
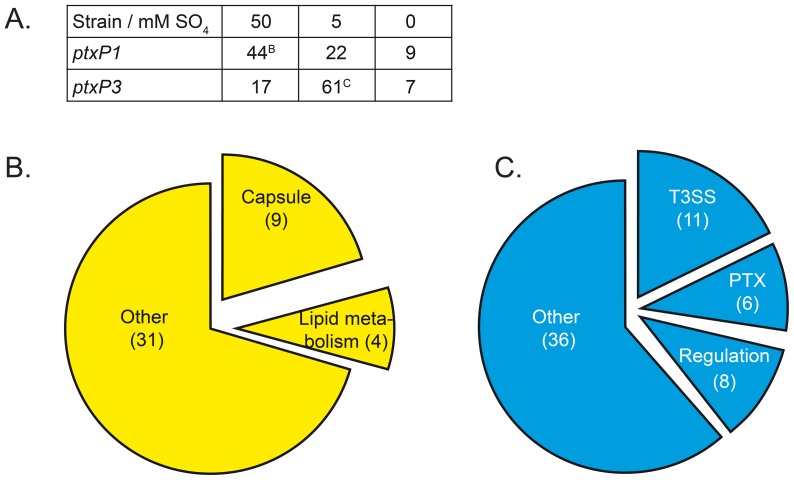
Genes differentially regulated between *B. pertussis* strain B1920 (*ptxP1*) and B1917 (*ptxP3*) upon sulfate-modulation. Sulfate was used to induce high (50 mM), medium (5 mM), and low (<0.02 mM, represented as 0 mM) sulfate conditions in a *ptxP3* and *ptxP1* strain. A) The number of genes expressed at least 3-fold higher between strains are indicated for each sulfate concentration. B&C) The pie charts subdivide the 44 genes expressed higher in the *ptxP1* strain as compared to the *ptxP3* strain under high sulfate conditions (B) and the 61 genes expressed higher in the *ptxP3* strain as compared to the *ptxP1* strain under medium sulfate conditions (C) into functional gene categories. The number of genes belonging to each category is numbered between brackets.

Under low sulfate conditions, the overall absolute gene expression levels were highly similar between the two strains. Only nine and seven genes were more than threefold higher expressed in the *ptxP1* and *ptxP3* strain, respectively, with no significant enrichment for genes of any particular functional category (Figure 3A and Table S6). In the previous section we showed that several genes involved in sulfate uptake and metabolism were differentially regulated by sulfate between the *ptxP1* and *ptxP3* strain. When looking at absolute expression levels under low sulfate conditions, seven sulfate genes were ≥3-fold higher expressed in the *ptxP3* strain, although only two were statistically significant (Table 1). The *cysB* gene, encoding the master regulator for *cys* gene expression, was also expressed at significant higher levels in the *ptxP3* strain, by 2.7 fold (Table 1).

Under medium sulfate conditions, the overall absolute gene expression levels were more distinct between the strains (Figure 3A and Table S6). Respectively, 22 and 61 genes were ≥3-fold higher expressed in the *ptxP1* and *ptxP3* strain. The higher expression of transcriptional regulators and virulence-associated genes by the *ptxP3* strain was the most prominent, with eight transcriptional regulators, one autotransporter gene (*vag8*), six genes for Ptx, and eleven genes for the T3SS toxin being expressed at higher levels in the *ptxP3* strain (Figure 3C and Table S6). In contrast, the genes expressed at higher levels in the *ptxP1* strain contained no obvious virulence-associated genes but predominantly housekeeping genes, in particular genes involved in leucine synthesis.

Under high sulfate conditions, the overall absolute gene expression levels were also distinct between the strains, with 44 and 17 genes being ≥3-fold higher expressed in the *ptxP1* and *ptxP3* strain, respectively (Figure 3A and Table S6). The 44 genes expressed higher in the *ptxP1* strain included a number of capsule genes and genes involved in lipid metabolism (Figure 3B and Table S6).

### Differences in gene expression between *ptxP1* and *ptxP3* strains in relation to DNA polymorphisms

The availability of the complete genome sequences of the two *B. pertussis* strains used in this study allows the association of gene expression differences to strain-specific DNA polymorphisms [23]. The expression levels of all genes associated with strain-specific polymorphisms can be found in Table S8. Of the 13 genes that are deleted in strain B1917, seven were expressed above the expression minima (see Text S1) in the B1920 strain, while 7 of the 18 genes absent in strain B1920 were expressed in the B1917 strain. Strain-specific transcriptional regulators may have a large impact on differential gene expression between strains. Indeed, both strains contain a unique transcriptional regulator (BP1963 in B1920 and BB1150 in B1917), both of which were expressed. With the exception of the two transcriptional regulators and the SNP in *ptxP*, no other polymorphism suggested underlying causes for the differences in gene expression between the two strains.

### Validation of sulfate-related differences in gene expression using multiple *ptxP1* and *ptxP3* strains

**Figure 4:**
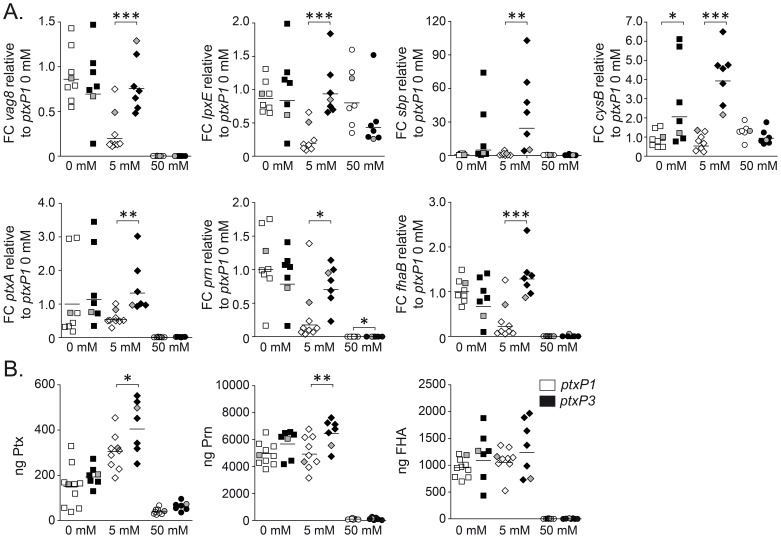
Validation of microarray data using multiple *B. pertussis ptxP1* and *ptxP3* strains. Sulfate was added to the culture medium to induce high (50 mM), medium (5 mM), and low (<0.02 mM, represented as 0 mM) sulfate conditions. (A) qRT-PCR data showing the relative expression level of *vag8*, *lpxE, sbp, cysB, ptxA, prn,* and *fhaB* between *ptxP1* strains (represented by white symbols) and *ptxP3* strains (represented by black symbols) grown under low (0 mM), medium (5 mM) and high (50 mM) sulfate conditions. The data are expressed as fold changes relative to the *ptxP1* strains grown under low sulfate conditions, with horizontal bars representing the geometrical mean, so that any sulfate-dependent effects on gene expression become apparent. B) Luminex data showing Ptx, Prn, and FHA protein expression in *ptxP3* and *ptxP1* strains grown under low (0 mM), medium (5 mM) and high (50 mM) sulfate conditions. Protein expression is expressed in absolute amounts (nanograms) with horizontal bars representing the geometrical mean. The expression values of the two strains that were used for microarray analysis (B1920 (*ptxP1*) and B1917 (*ptxP3*)) are depicted in grey. Asterisks indicate a statistically significant difference between the groups as determined by an unpaired Student's t-test: * *P* value <0.05, ** *P* value <0.005, *** *P* value <0.0005.

Microarray analysis suggested that *ptxP3* strains are less sensitive to sulfate-mediated suppression of virulence gene expression, resulting in a higher expression of virulence genes under medium sulfate conditions. To determine whether this is a general lineage-dependent phenotypical difference, 16 non-epidemiologically related *ptxP1* (n = 9) and *ptxP3* (n = 7) strains (Table 3) were cultured *in vitro* in the presence of low, medium, and high sulfate and analyzed by qPCR for a number of genes (Figure 4A). qPCR analysis showed that not only *vag8, lpxE*, *sbp, cysB*, but also the three major vaccine antigens *ptxA*, *prn*, and *fhaB* were significantly higher expressed in the *ptxP3* strains under medium sulfate conditions (Figure 4A). Since transcriptional activity does not necessarily correlate with the amount of protein produced, we used a Luminex assay to quantify the amount of Ptx, Prn, and FHA protein produced by these strains. Similar to qPCR, we found that *ptxP3* strains produced more Ptx and Prn than the *ptxP1* strains, but not FHA (Figure 4B).

**Table 3:** Characteristics of the Dutch *Bordetella pertussis* strains used in this study.

				Alleles				
Strain	Year isolation	Age patient (in months)	Serotype	*ptxA*	*ptxP*	*prn*	*fim3*	*fim2*
B0638	1996	96	3	*ptxA1*	*ptxP1*	*prn3*	*fim3-1*	*fim2-1*
B0888	1991	9	3	*ptxA1*	*ptxP1*	*prn3*	*fim3-1*	*fim2-1*
B1878	2000	45	2	*ptxA1*	*ptxP1*	*prn2*	*fim3-1*	*fim2-1*
B1920	2000	9	3	*ptxA1*	*ptxP1*	*prn2*	*fim3-1*	*fim2-1*
B2414	2002	25	3	*ptxA1*	*ptxP1*	*prn1*	*fim3-4*	*fim2-2*
B2968	1987	unknown	2,3	*ptxA2*	*ptxP1*	*prn1*	*fim3-1*	*fim2-1*
B3124	2007	1	2	*ptxA1*	*ptxP1*	*prn2*	*fim3-1*	*fim2-1*
B3214	2008	160	2	*ptxA1*	*ptxP1*	*prn2*	*fim3-1*	*fim2-1*
B3379	2009	43	2	*ptxA1*	*ptxP1*	unknown	*fim3-1*	*fim2-1*
B1917	2000	44	3	*ptxA1*	*ptxP3*	*prn2*	*fim3-2*	*fim2-1*
B2584	2003	2	3	*ptxA1*	*ptxP3*	*prn2*	*fim3-1*	*fim2-1*
B3034	2005	1	3	*ptxA1*	*ptxP3*	*prn2*	*fim3-2*	*fim2-1*
B3230	2008	21	3	*ptxA1*	*ptxP3*	*prn2*	*fim3-2*	*fim2-1*
B3448	2010	1	2	*ptxA1*	*ptxP3*	*prn2*	*fim3-1*	*fim2-1*
B3928	2012	13	2	unknown	*ptxP3*	unknown	*fim3-1*	*fim2-1*
B3956	2012	2	2	*ptxA1*	*ptxP3*	*prn2*	*fim3-1*	*fim2-1*

## Discussion

The worldwide selective sweep of the *ptxP3* lineage and its link to recent pertussis epidemics in some countries clearly emphasizes the importance of studies on the molecular mechanisms underlying these phenomena [13], . Although the success of a particular lineage is also determined by host immunity factors, here we focused on the molecular characterization of the *ptxP3* lineage. Until recently, the only distinctive phenotypes described for *ptxP3* strains was a higher Ptx production and enhanced respiratory colonization [13], [30]. Here we identify additional phenotypic differences between *ptxP1* and *ptxP3* strains which may have contributed to its global spread. In previous comparative genomic studies we showed that *ptxP1* and *ptxP3* strains have different SNPs in a number of virulence-associated genes, differences in pseudogenes, as well as differences in gene content [23], [34]. A recent transcriptional comparison of *ptxP1* and *ptxP3* strains under non-modulating conditions indicated that multiple virulence-associated genes are expressed at slightly higher levels in *ptxP3* strains as compared to *ptxP1* strains [30]. Here, we compared sulfate-dependent expression profiles between these strains, with the rationale that sulfate affects the expression of all major *B. pertussis* virulence factors [28]. This approach identified several additional differences between the *ptxP1* and *ptxP3* strains, including sulfate-dependent differences in expression levels of a number of important virulence genes and a different sensitivity for sulfate-mediated regulation. Conceivably, the two are interconnected.

The most pronounced phenotypic difference revealed by microarray analysis was that *ptxP1* and *ptxP3* strains respond differently to sulfate mediated-regulation. Based on genome annotations and BLAST searches we identified 30 genes that are likely involved in sulfate metabolism, nine of which were differentially regulated between the *ptxP1* and *ptxP3* strain. The sulfate genes included genes involved in uptake and metabolism of sulfate, cysteine, methionine and taurine. Interestingly, taurine is one of the most abundant sources of sulfate in the host, comprising 0.1% of the total human body weight [35]. In general, the sulfate genes were expressed at higher levels by the *ptxP3* strain under low sulfate conditions compared to the *ptxP1* strain (Table 1). For instance, the *sbp* gene, which facilitates transport of external sulfate into the cell [36], [37], showed a 9-fold higher level of gene expression in the *ptxP3* strain. Furthermore, nine other sulfate genes were expressed at two- to four-fold higher level in the *ptxP3* strain under low sulfate conditions, although not all values reached statistical significance. The increased expression of sulfate genes in the *ptxP3* strain may be explained by the observation that the positive master regulator for *cys* gene expression, *cysB* [38], was more highly expressed in *ptxP3* strains than *ptxP1* strains under low and medium sulfate conditions. It is tempting to speculate that the expression profile of these genes contributes to the reduced sensitivity of *ptxP3* strains to sulfate-mediated suppression of known Bvg-regulated genes as shown in this work. For instance, we found that *ptxP3* strains in particular, and to a lesser extent *ptxP1* strains, expressed higher levels of Ptx in the presence of 5 mM sulfate (Figure 4), whilst *ptx* genes have been described to be suppressed under this condition [39], [40]. This suggests that the protein expression pattern of Ptx in *ptxP3* strains more accurately reflects a Bvg^i^-phase protein than the classical Bvg^+^ profile. Recently, it was described that the BvgS sensor molecule is active by default and is only inhibited when sulfate or other negative modulators bind to the Venus Flytrap (VFT) 2 region in the periplasmic domain of this sensor molecule [41]. Whilst the concentration of free sulfate in the respiratory tract is low (0.6 mM; [42]), infection may increase sulfate concentration locally, e.g. through desulfation of sulfated host proteins [43], potentially through pertussis proteins containing a sulfatase domain (BP1635, BP1654, BP2327, and BP3136) of which ORF BP3136 was expressed above the expression minima in both strains. Interestingly, the *wcbQ* gene (BP1654) encoding a capsular polysaccharide biosynthesis protein with a sulfatase domain, was expressed exclusively and at 4.5 fold higher levels in the *ptxP1* strain under low and medium sulfate conditions, as compared to the *ptxP3* strain (Table S7). Extracellular sulfate can diffuse freely through the outer membrane into the periplasmic region and bind to the VFT2 region of BvgS [44]. As such, *ptxP3* strains may benefit from the lack of *wcbQ* expression and the increased expression of the *sbp*, *cysT*, and *cysW* sulfate transport genes, as this might lower periplasmic sulfate levels. It is therefore conceivable that the concentration of free sulfate in the periplasmic space is lower in the *ptxP3* strain compared to the *ptxP1* strain. Thus, at equal concentrations of extracellular sulfate, less suppression would occur in the *ptxP3* strain. It is questionable whether this effect is transduced by the BvgASR system only, as only a limited number of known Bvg-regulated genes were affected. Here, we speculate that sulfate may regulate *B. pertussis* (virulence) genes via a second route, possibly comprised of a sensory transduction system. Indeed under medium sulfate conditions eight transcriptional regulators were more highly expressed in the *ptxP3* strain compared to the *ptxP1* strain (Figure 3C, Table S6). This hypothetical second regulon includes both Bvg-regulated genes and genes which are regulated independent of the Bvg-system. One possibility to test this hypothesis would be to examine the response of Bvg-phase locked mutants to different sulfate concentrations.

The higher expression level of *cys* genes in the *ptxP3* strain may also suggest that these strains are more resistant to Reactive Oxygen Species (ROS), as several publications have found a link between the two [45], [46]. However, this remains to be investigated.

Another interesting gene which was differentially regulated between the *ptxP1* and *ptxP3* strains was *lpxE*, encoding a lipid A-1 phosphatase. The *lpxE* gene was identified as a HSR gene specifically in the *ptxP3* strain and was also expressed at four-fold higher levels in the *ptxP3* strains under medium sulfate conditions. The lipid A-1 phosphatase encoded by this gene is responsible for selectively dephosphorylating the 1-position of lipid A [47]. It is well established that the presence of phosphate groups on the lipid A moiety of the lipo-oligosaccharide (LOS) is essential for the endotoxic activity of LOS [47], [48]. Since LOS lacking the 1-phosphate group are recognized less efficiently by the Toll-like receptor 4 (TLR4)/myeloid differentiation factor (MD-2) receptor complex of the mammalian innate immune system, they induce a weaker proinflammatory cytokine response [49]. This effect has been described for a number of bacterial pathogens. For instance, in *Salmonella typhymurium*, genomic introduction of the *lpxE* gene from *Francisella tularensis* led to a clear reduction of virulence in a mouse model [50]. Furthermore, the human pathogen *Helicobacter pylori* uses dephosphorylation of both the 1- and 4-phosphate to hide itself from recognition by the innate immune system, allowing the pathogen to survive in the gastric mucosa [51]. Whether the *lpxE* gene facilitates a similar function in *B. pertussis* remains unknown. For *B. pertussis* it is known that the 1-phosphate group of lipid A can be substituted by glucosamine in a Bvg-regulated manner and that this modulates hosts immune defenses [52], [53]. However, this substitution is strain-specific, and has been studied exclusively in routinely used laboratory strains [54]. Consequently, the lipid A composition of currently circulating *B. pertussis* strains remains unknown. Nonetheless, it is tempting to speculate that the differential sulfate-dependent regulation of the *lpxE* gene in *ptxP1* and *ptxP3* strains has an influence on the endotoxic activity and immune modulating capacity of these strains.

Another difference between the *ptxP1* and *ptxP3* strains identified in this study was the limited overlap (51%) in genes being induced by high sulfate (HSI genes, Figure 2D). However, this in itself is not unexpected, as previous work also showed significant gene expression differences in Bvg^−^ locked *B. pertussis* strains [55]. This heterogeneity in HSI gene profiles may indicate a lack of purifying selection, which further supports the idea that the Bvg^−^ phase of *B. pertussis* is an evolutionary remnant [56]. In *B. bronchiseptica*, the evolutionary ancestor of *B. pertussis*, the Bvg^−^ phase is assumed to be important for (*ex vivo*) survival under nutrient-limiting conditions [39], [57]. However, *B. pertussis* has evolved into an obligate human pathogen which does not require an environmental niche [58].

In a previous study we showed that *ptxP3* strains grown on plates produce more Ptx than *ptxP1* strains [13]. We explored this difference further here using liquid cultures and observed that the largest difference in Ptx expression was observed at medium sulfate concentrations. We did not observe increased Ptx expression under non-modulating conditions as in our previous study. However, this might be related to the different growth media used (plates versus chemically defined liquid medium) and/or the growth phase at which the bacteria were collected (after 3 days on plate versus during mid-log growth). A novel finding was the higher expression of T3SS proteins and of the autotransporters Prn and Vag8 by *ptxP3* strains under medium sulfate conditions. Slightly increased levels (fold change 1.2–1.8) of T3SS and Vag8 have also been reported by others under non-modulating conditions [30]. The difference in Ptx expression may be explained by the mutation in the Ptx promoter region, as suggested previously [13]. However, no mutations were found in the ORFs or promoter regions of the Prn, Vag8, and T3SS genes, suggesting that polymorphisms in other genes may (also) be involved in their transcriptional regulation. All *ptxP3* strains analyzed to date contain a deletion encompassing BP1948–1966 [34] and it is possible that the deletion of these genes plays a role in the differential regulation of these genes. Conversely, the *ptxP3* strain B1917 also contains genes (BB1140–BB1158) that are absent from *ptxP1* strain B1920, including two transcriptional activators (BB1141 and BB1150), which may also contribute to the observed differences. The expression phenotype of these three important virulence factors in the *ptxP3* strains at medium sulfate concentrations, is significant as all three are involved in suppression and modulation of the host immune response. In this sense, Ptx is the most versatile virulence factor, as it is able to intoxicate alveolar macrophages (AMs) [59], inhibit the mucosal recruitment of immune cells (such as AMs, neutrophils, and T cells) [60]–[62], modulates the cellular immune response [63], and suppresses serum antibody responses [64], [65]. Furthermore, T3SS represents a multi-component secretion machinery used by a wide variety of gram-negative bacteria to secrete effectors directly into the cytosol of host cells and interfere with host cell functioning. In *B. pertussis*, two proteins have been identified as T3SS effectors: BteA and BopN. BteA is a cytotoxin that induces a rapid non-apoptotic death in host epithelial cells [66] while BopN modulates cellular immune responses [67]. Additionally, the autotransporter Vag8 mediates the binding of human C1 esterase inhibitor on the bacterial surface and thereby confers resistance to complement-mediated killing [31]. Given the important virulence properties of these proteins, *ptxP3* strains may benefit from their increased expression, although a direct link to enhanced immune suppression remains to be established.

Taken together, comparative transcriptional profiling of a *ptxP1* and a globally emerged *ptxP3* strain of *B. pertussis* provided novel insights into sulfate-mediated modulation of the *ptxP3* lineage and should stimulate research into the role of sulfate in the pathogenesis of *B. pertussis*. Although it is tempting to focus on specific genes, the overall increased expression of multiple virulence factors in the *ptxP3* strain may be more important, as this suggests that this strain is in a higher state of virulence, which may allow for better transmission among immune hosts. Thus both antigenic divergence with vaccine strains [11] and increased immune suppression may have contributed to the global spread of *ptxP3* strains.

## Materials and Methods

### Ethics Statement

The only patient material collected were strains which are not collected by us and not specifically for this study. The strains were collected by Medical Microbiology Laboratories from patients suspected of whooping cough and sent to the RIVM in the context of routine surveillance (as required by law). The strains are sent to the RIVM for confirmation of clinical diagnosis, species determination and subtyping. Strictly anonymized patient information is included, which is limited to age, sex and postal code. For this type of surveillance ethical evaluation or patient consent are not required. The strains have been used in previous studies [22].

### Bacterial strains and growth conditions

Two recent *B. pertussis* strains isolated from patients in the Netherlands in 2000 were selected to study sulfate-modulated gene expression: B1920 (*ptx*P1) and B1917 (*ptx*P3) [13] (Table 3). The effect of sulfate on gene expression was extended with eight and six additional non-related *ptxP1* and *ptxP3* strains, respectively, isolated from patients from different geographical regions in the Netherlands between 1987 and 2012 (Table 3). *B. pertussis* strains were grown on Bordet-Gengou agar plates supplemented with 15% sheep blood (Tritium Microbiology, Eindhoven, The Netherlands) and incubated for four days at 37°C. Liquid cultures were grown overnight in chemically defined THIJS medium [68] supplemented with 0.2 mg/ml Heptakis-cyclodextrin (Sigma) and then re-inoculated into pre-warmed medium at an optical density at 620 nm (OD_620_) of 0.075. For modulation of the *BvgASR* regulatory system, magnesium sulfate was added to the cultures at a final concentration of 5 and 50 mM to induce medium and high sulfate conditions respectively. In the absence of additional sulfate, the concentration of free sulfate in THIJS medium was <0.02 mM as determined using the QuantiChrom sulfate assay kit (BioAssay Systems), thereby inducing low sulfate conditions. Cultures were grown at 37°C until a mid-log OD_620_ of 0.5 to 0.6, at which point the bacteria were harvested for RNA isolation and Luminex analysis as described below.

### RNA isolation and qRT-PCR

Aliquots of 5 ml mid-log culture were mixed with two volumes of RNA Protect Bacteria Reagent (Qiagen). Total RNA was extracted using the RNeasy Mini kit (Qiagen) and contaminating genomic DNA was subsequently removed by DNase treatment (DNAfree, Ambion). DNA-free total RNA (125 ng) was reverse transcribed using 300 ng of random hexamers (Invitrogen), and Superscript III reverse transcriptase (200U, Invitrogen) in 1x First-Strand buffer, 10 mM DTT, and 0.5 mM dNTPs. To confirm the absence of genomic DNA, control reactions were carried out without reverse transcriptase. Relative amounts of *ptxA* (BP3783), *bipA* (BP1112), *kpsT* (BP1624), *prn* (BP1054), *fim3* (BP1568), *fhaB* (BP1879), *vag8* (BP2315), *lpxE* (BP0835), *sbp* (BP0966), and *cysB* (BP2416) transcripts were determined by quantitative real-time-PCR (qRT-PCR) using the SYBR green technology with the primers listed in Table S1 on a 7500 Fast real-time PCR system (PE Applied Biosystems) according to the manufacturer's instructions. The relative quantification ΔΔCt method was used to compare expression levels between the different strains and sulfate-conditions [69]. The *recA* (BP2546) amplicon was used as internal control for normalization of data.

### Microarray expression profiling

Five µg of total RNA was labeled by a method adapted from Ouellet *et al*. [70], as described by de Vries and coworkers [71]. Two μg of labeled cDNA was applied to a 12x135K custom design NimbleGen array. Overnight hybridization at 42°C and subsequent washing of arrays was performed according to the manufacturer's instructions. The NimbleGen array contained 1–8 probes for all coding sequences (CDS) with an average coverage of 7.5 probes per CDS and pseudogene, 15 bp overlapping tiling probes covering both strands of the intergenic regions, and 7,304 random probes with a similar length distribution and GC content as the experimental probes. The array design was based on the genome sequence of *B. pertussis* Tohama I [72], supplemented with additional *B. bronchiseptica* CDS and intergenic sequences that are present in the *B. pertussis* strains used in this study [23]. Array images were acquired with a NimbleGen MS200 scanner, and images were processed with NimbleScan software. Normalized expression data was analyzed by ArrayStar (DNASTAR, Madison, WI, USA) using the Robust Multiarray Analysis (RMA) algorithm for background correction and quantile normalization [73]. For the identification of sulfate-modulated genes the raw expression data under low, medium, and high sulfate was normalized individually for each strain. To identify absolute differences in gene expression between the *ptxP1* and *ptxP3* strain, normalization was performed using the raw expression data of both strains under all sulfate conditions. The latter normalized expression values can be found in Table S7. Log_2_ transformed signals were used to generate kernel density plots using a Gaussian model with stepwise increasing bandwidth until a single local minimum was found between the distributions of background signal and gene expression. Positions where the first derivative of the density traverses from values below to values above zero were considered local minima. The corresponding expression value is the value closest to the minimum between the peaks of expressed and non-expressed genes and was therefore considered as a cutoff value to determine whether a gene was expressed or not. Further validation of the transcriptomic dataset is described in Text S1 and Table S2.

A moderated Student t-tests using Benjamin Hochberg correction with a cutoff P-value of 0.05 was used to compare the mean gene expression values. Genes were excluded if the expression was low (signal intensity <500) in both conditions that were compared. High sulfate repressed (HSR) genes and high sulfate induced (HSI) genes were defined as those genes which were expressed at a level at least three times higher or lower, respectively, in the absence of sulfate compared to growth in the presence of 50 mM sulfate. Medium sulfate induced (MSI) or medium sulfate repressed (MSR) genes were defined as those genes which were significantly up- or down regulated respectively by at least three-fold when bacteria were grown in the presence of 5 mM sulfate compared to <0.02 mM sulfate. Functional class distribution was assessed using the Institute for Genomic Sciences (IGS) classification (http://www.igs.umaryland.edu/) and the protein localization was predicted using PSORTb v3.0 [74]. Enrichment for functional class and localization was assessed using the Fishers exact (one-tail) test and considered significant at a p-value <0.05. Enrichment for specific sets of genes were assessed using the Database for Visualization and Integrative Discovery (DAVID) [75].

### Microarray data

All microarray data have been deposited in NCBI's Gene Expression Omnibus (GEO) database (www.ncbi.nlm.nih.gov/geo/) and are accessible through GEO Series accession number GSE49385 (http://www.ncbi.nlm.nih.gov/geo/query/acc.cgi?acc=GSE49385).

### Multiplex immunoassay

One ml aliquots of mid-log cultures were heat-inactivated (HI) for 30 min at 56°C and used in a multiplex immunoassay (MIA) for the quantification of pertussis toxin (Ptx), filamentous hemagglutinin (FHA), and pertactin (Prn) by a method adapted from van Gageldonk *et al*. [76]. Purified monoclonal anti-Ptx (Pem 9), anti-FHA (29E7), and anti-Prn (Pem 85) antibodies (kindly provided by Dr. Guy A.M. Berbers) were coupled to activated carboxylated microspheres (Bio-Rad Laboratories) using a two-step carbodiimide reaction [77]. Bead regions were carefully chosen not to be directly adjacent to each other: 9 (anti-Prn), 24 (anti-FHA), 33 (anti-Ptx). A solution of 500 µl of carboxylated microspheres (6.25×10^6^ beads) was washed once by centrifugation (12,000×g for 2 min) with 250 μl dH_2_O and resuspended in 200 μl 0.1 M Monobasic Sodium Phosphate, pH 6.2 and activated by addition of 25 µl of N-hydroxy-sulfosuccinimide (sulfo-NHS. Pierce) 50 mg/ml and 25 µl of 1-ethyl–3-(-3-dimethylaminopro-pyl)-carbodiimide hydrochloride (EDC. Pierce) 50 mg/ml. The solution was incubated for 20 min at room temperature (RT), in the dark under constant rotation at 25 rpm. The activated microspheres were washed twice with 0.05 M 2[N-Morpholino] ethanesulfonic acid (MES, Sigma-Aldrich) pH 5.0 by centrifugation (12,000×g for 2 min) and resuspended in 1.25 ml 0.05 M MES pH 5.0 with a monoclonal to bead ratio of 12.5 µg/6.25×10^6^ activated beads for anti-Ptx and anti-Prn, and 62.5 µg/6.25×10^6^ activated beads for anti-FHA. The beads were incubated for 2 h at RT in the dark under constant rotation at 25 rpm. Subsequently, the beads were washed three times with PBS containing 0.05% Tween 20 (PBST) and stored in 1 ml PBST at 4°C in the dark until used.

HI-cultures were diluted 1/50 in PBS pH 7.2 containing 0.1% (v/v) Tween-20 and 1% (w/v) BSA (assaybuffer) before use. Ptx obtained from Kaketsuken (Kumamoto, Japan), FHA obtained from SmithKline Beecham, and Prn obtained from the Pediacell vaccine of Sanofi Pasteur MSD were used as standards in a concentration range of 243, 81, 9, 3, and 1 ng/ml in assaybuffer. Each dilution of the standard and culture samples (25 µl) was mixed with an equal volume of the conjugated microspheres (Ptx, Fha, Prn; 4000 beads/region/well) in a 96-well Multiscreen HTS filter plate (Millipore Corporation, Billerica, MA). Plates were incubated for 60 min at RT in the dark on a plate shaker at 600 rpm. Blanks were included on every plate. The beads were collected by filtration using a vacuum manifold and washed three times with 100 µl assaybuffer. A volume of 50 µl of a 1/200 dilution of Human Pertussis Antiserum (WHO International Standard Pertussis Antiserum 06/140 NIBSC, Hertfordshire, UK) was added to each well and the plate was incubated in the dark for 20 min with continued shaking. The beads were collected and washed as described above. To each well 100 µl of a 1/200 dilution of R-phycoerthyryn (R-PE)-conjugated goat anti-human IgG (Fc_y_ Fragment specific, Jackson ImmunoResearch Laboratories Inc, Westgrove, Pa) was added and the plate was incubated for 20 min in the dark with continued shaking. The beads were collected and washed in PBS containing 0.05% Tween as described above. Finally the beads were resuspended in 125 µl PBS and shaken in the dark before analysis with a Bio-Plex 100 system in combination with Bio-Plex Manager software version 4.1.1 (Bio-Rad Laboratories, Hercules, CA). For each analyte, median uorescent intensity (MFI) was converted to ng/ml by interpolation from a 5-parameter logistic standard curve (log–log) for every bead region/standard.

## Supporting Information

Figure S1 **Sulfate-mediated** ***fim3*** **expression in** ***B. pertussis*** **strain B1920 (** ***ptxP1*** **) and B1917 (** ***ptxP3*** **).** Sulfate was added to the culture medium to induce high (50 mM), medium (5 mM), and low (<0.02 mM, represented as 0 mM) sulfate conditions. qRT-PCR data shows the relative expression level of *fim3* expressed as fold changes relative to the high sulfate condition, with the values being the mean of four biological replicate cultures. Asterisks indicate a statistically significant difference between the groups as determined by Student's t-test with Welch's correction: * *P* value <0.05, ** *P* value <0.005, *** *P* value <0.0005.
(TIF)

Text S1 **Validation transcriptomic datasets.**
(DOCX)

Table S1 **qRT-PCR primers used in this study.**
(XLSX)

Table S2 **Pearson correlation analysis of the microarray datasets of the** ***ptxP1*** **and** ***ptxP3 B. pertussis*** **strains grown in the presence of different sulfate concentrations.**
(XLSX)

Table S3 **Identification of high sulfate induced and repressed genes in** ***ptxP1*** **and** ***ptxP3*** **strains.**
(XLSX)

Table S4 **Identification of** ***ptxP1*** **and** ***ptxP3*** **strain-specific HSI and HSR genes.**
(XLSX)

Table S5 **Identification of medium sulfate induced or repressed genes in the** ***ptxP1*** **and** ***ptxP3*** **strain.**
(XLSX)

Table S6 **Genes that show significantly different levels of expression in** ***ptxP1*** **and** ***ptxP3*** **strains in the presence of low, medium, and high sulfate.**
(XLSX)

Table S7 **Absolute expression levels of genes in** ***ptxP1*** **and** ***ptxP3*** **strains according to microarray analyses.**
(XLSX)

Table S8 **Sulfate-regulated expression levels of genes with strain-specific DNA polymorphisms in** ***ptxP1*** **and** ***ptxP3*** **strains.**
(XLSX)
