# Correction: Differentially Expressed Genes in *Bordetella pertussis* Strains Belonging to a Lineage Which Recently Spread Globally

**DOI:** 10.1371/annotation/1b2cfe52-aaea-4148-aa72-a74c78550192

**Published:** 2014-01-16

**Authors:** Daan de Gouw, Peter W. M. Hermans, Hester J. Bootsma, Aldert Zomer, Kees Heuvelman, Dimitri A. Diavatopoulos, Frits R. Mooi

The name of the first author was incorrectly represented in the Citation. The correct Citation is: 

de Gouw D, Hermans PWM, Bootsma HJ, Zomer A, Heuvelman K, et al. (2014) Differentially Expressed Genes in Bordetella pertussis Strains Belonging to a Lineage Which Recently Spread Globally. PLoS ONE 9(1): e84523. doi:10.1371/journal.pone.0084523.

