# Supplementary figures and images for: Differentially Expressed Genes in Bordetella pertussis Strains Belonging to a Lineage Which Recently Spread Globally
Source: PLoS One. 2014 Jan 8;9(1):e84523. doi: 10.1371/journal.pone.0084523 (PMC3885589; doi:10.1371/journal.pone.0084523)

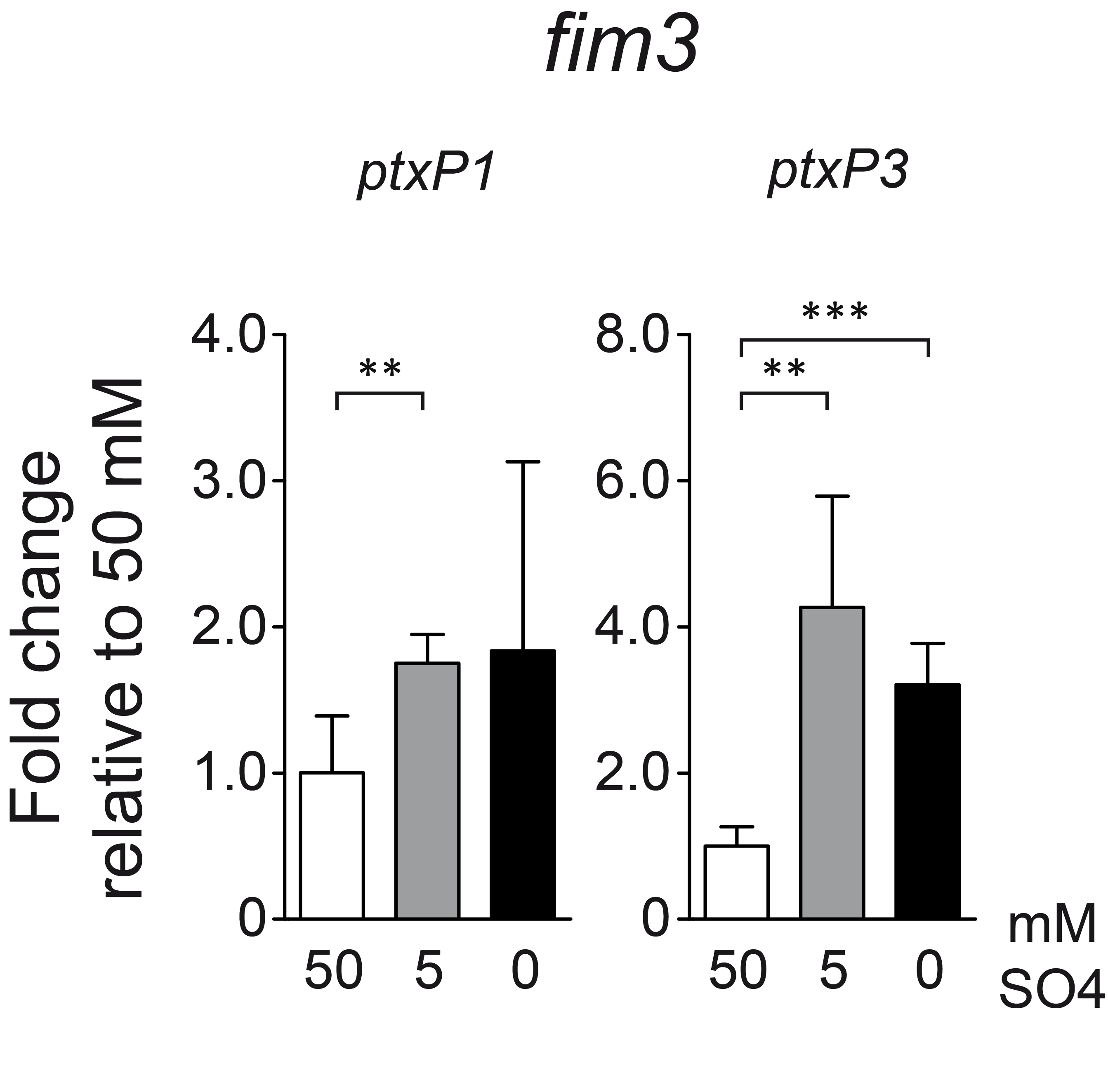

Supplement: Figure S1 — Sulfate-mediated fim3 expression in B. pertussis strain B1920 ( ptxP1 ) and B1917 ( ptxP3 ). Sulfate was added to the culture medium to induce high (50 mM), medium (5 mM), and low (<0.02 mM, represented as 0 mM) sulfate conditions. qRT-PCR data shows the relative expression level of fim3 expressed as fold changes relative to the high sulfate condition, with the values being the mean of four biological replicate cultures. Asterisks indicate a statistically significant difference between the groups as determined by Student's t-test with Welch's correction: * P value <0.05, ** P value <0.005, *** P value <0.0005. (TIF) [file pone.0084523.s001.tif]
