# Supplementary material for: Differentially Expressed Genes in Bordetella pertussis Strains Belonging to a Lineage Which Recently Spread Globally
Source: PLoS One. 2014 Jan 8;9(1):e84523. doi: 10.1371/journal.pone.0084523 (PMC3885589; doi:10.1371/journal.pone.0084523)
Supplement: Text S1 — Validation transcriptomic datasets. (DOCX) [file pone.0084523.s002.docx]

**Supplemental Text S1**

*Validation transcriptomic datasets*

At the mRNA level, 74% of the 3552 genes present in the *ptxP3* strain and 79% of the 3548 genes present in *ptxP1* were found to be expressed based on cutoffs defined by kernel density plots. Pearson correlation analysis was used to determine the correlation in mRNA abundance between the sulfate conditions of the *ptxP1* and *ptxP3* strain. The obtained correlation coefficient varied between 0.00 and 1.00, in which a value of 1.00 indicates a perfect correlation and a value of 0.00 indicates the absolute lack of correlation. Log2 values of the mean signal intensities (SI) of all genes on the microarray were used in this analysis and revealed excellent correlation (0.96-0.98) between the two strains for each sulfate-condition (Table S2). Furthermore, as expected, within strains we observed a strong correlation between the low and medium sulfate condition (0.99) and a weaker correlation between the low and high sulfate condition (0.87-0.90).
